# Prognostic Factors of Pulmonary Metastasectomy for Soft Tissue Sarcomas Arising in the Trunk Wall and Extremities

**DOI:** 10.3390/cancers14143329

**Published:** 2022-07-08

**Authors:** Shizuhide Nakayama, Eisuke Kobayashi, Jun Nishio, Yu Toda, Masaya Yotsukura, Shun-Ichi Watanabe, Takuaki Yamamoto, Akira Kawai

**Affiliations:** 1 Department of Musculoskeletal Oncology and Rehabilitation Medicine, National Cancer Center Hospital, Tokyo 104-0045, Japan; n.shizuhide@gmail.com (S.N.); yutoda2@ncc.go.jp (Y.T.); akawai@ncc.go.jp (A.K.); 2 Department of Orthopaedic Surgery, Faculty of Medicine, Fukuoka University, Fukuoka 814-0180, Japan; yamamotot@fukuoka-u.ac.jp; 3 Section of Orthopaedic Surgery, Department of Medicine, Fukuoka Dental College, Fukuoka 814-0193, Japan; nishio@college.fdcnet.ac.jp; 4 Department of Thoracic Surgery, National Cancer Center Hospital, Tokyo 104-0045, Japan; mayotsuk@ncc.go.jp (M.Y.); syuwatan@ncc.go.jp (S.-I.W.)

**Keywords:** pulmonary metastasis, pulmonary metastasectomy, prognostic factor, disease-specific survival

## Abstract

**Simple Summary:**

Pulmonary metastasectomy (PM) is often performed in sarcoma patients with resectable oligo-metastases in the lungs. Although there have been several studies on PM for sarcomas, few have analyzed only soft tissue sarcomas (STSs) arising in the trunk wall and extremities, and many of these studies are older. Therefore, it would be of interest to confirm the outcomes of PM for STS in recent years, when systemic treatment has advanced, in accordance with the latest WHO classification. In the present study, we investigated our recent results of PM for STSs arising in the trunk and extremities, and analyzed the prognostic factors and safety of PM.

**Abstract:**

Although there is no evidence from prospective randomized controlled trials to support this practice, pulmonary metastases of sarcomas are often treated surgically if they are resectable. The purpose of this retrospective study was to evaluate the prognostic factors and outcome of pulmonary metastasectomy (PM) for soft tissue sarcomas (STSs) arising in the trunk wall and extremities in 66 consecutive patients. Prognostic factors associated with disease-specific survival after PM were evaluated using univariate and multivariate analyses. The patients included 38 men and 28 women, with a median age of 49 years. The median disease-specific survival after PM was 48 months, and the 5-year survival rate was 45%. No major perioperative complications occurred. Disease-free interval (<12 months), size of largest lung lesion (≥20 mm), and non-curative resection were independent prognostic factors in multivariate analysis. PM was effective in selected patients with pulmonary metastases from STSs arising in the trunk wall and extremities. Disease-free interval, maximum size of metastases, and resectability were identified as prognostic factors.

## 1. Introduction

Soft tissue sarcomas (STSs) are rare and heterogeneous malignancies of mesenchymal origin. STSs metastasize predominantly to the lungs, and pulmonary metastases are found in 17.8–25.2% of STSs [1,2,3,4]. Despite a lack of evidence from randomized controlled trials, surgery has been the mainstay of treatment for sarcoma with resectable oligo-metastases in the lungs over the past few decades [5,6]. Furthermore, because of its rarity and the unethical implications of randomization for such an aggressive disease, randomized controlled trials are considered difficult. Although the efficacy of pulmonary metastasectomy (PM) has not been proved at a high level of evidence, it is considered the only treatment that has the potential to cure the metastatic disease of STSs. Five-year survival rates ranging from 18% to 52% have been reported for STS patients with resectable metastases [1,4,7,8,9,10,11,12,13,14,15,16,17]. Several reports have shown that surgical treatment of pulmonary metastases of STSs prolongs survival, with median survival after PM of 19–38.9 months in the surgical group and 8–10.5 months in the non-surgical group [3,18,19].

Various prognostic factors have been proposed to influence overall survival rates after PM for STSs, including disease-free interval (DFI), resectability, histological subtype and grade of primary tumors, number and maximum size of metastatic nodules, and age [1,4,7,8,9,10,11,12,14,15,16,17,20,21]. However, most studies have simultaneously analyzed STSs arising from parenchymal organs, retroperitoneum, mediastinum, or head and neck [1,4,7,8,9,10,11,12,16,17,20,21], and few have examined STSs originating in the trunk wall and extremities, which are usually treated with a curative margin [3,14,15,19]. The classification of STSs has changed gradually in recent years because of the emergence of molecular-based histological subtypes [22]. Therefore, it is important to analyze clinical outcomes based on the latest classifications. The recent emergence of new therapeutic agents for STS may affect the prognosis after PM [23,24].

The number and size of tumors that can be controlled by PM, alongside the indications for perioperative chemotherapy, are not yet clear. This study was designed to clarify these issues. The purpose of this retrospective study was to identify prognostic factors for PM in STSs arising in the trunk wall and extremities and to evaluate the safety of PM.

## 2. Materials and Methods

This study was conducted in accordance with the Declaration of Helsinki. Approval of this study was granted by the Institutional Review Board at the National Cancer Center Hospital (NCCH, Tokyo, Japan), (Approval ID: 2021-325), and the requirement for written informed consent from each patient was waived. From January 2007 to January 2021, all consecutive patients who underwent curative-intent PM for STSs in NCCH were analyzed. Patients whose metastatic tumors originated from parenchymal organs, mediastinum, retroperitoneum, and head and neck were excluded.

The decision to perform PM was based on the proposals of Thomford et al. and multidisciplinary discussion in our institution about treatment options [25]. The principal conditions for PM were as follows: the patient could tolerate the surgical intervention; the primary malignancy was well-controlled; there was no metastasis other than in the lungs; the pulmonary metastasis was located unilaterally in the lungs. However, there were exceptions to these rules, including some cases of bilateral oligo-metastases that underwent PM following multidisciplinary discussion. Pulmonary resection was performed via the minimally invasive open approach, in combination with the use of a thoracoscope [26]. In most cases, skin incisions of 5–8 cm for thoracotomy and 1.5 cm for a thoracoscope were made. The type of pulmonary resection was selected depending on the size and location of the metastatic lesions. In case of bilateral lung metastases, a two-stage procedure was performed at the discretion of the thoracic surgeon.

Collected data included: demographic characteristics (age and sex); primary tumor characteristics (histopathology and site); DFI; timing of pulmonary metastasis; metastatic characteristics (number, distribution, and maximum diameter of nodules); type of lung resection; administration of adjuvant chemotherapy before and after PM; complications of PM (Clavien–Dindo Classification [27]); postoperative hospital stay and outcome. Histological diagnosis was based on the WHO Classification of Tumours of Soft Tissue and Bone (5th edition, IARC Press, Lyon, France) [22]. DFI was defined as the interval between curative primary surgery and diagnosis of pulmonary metastasis.

Disease-specific survival (DSS) was used as the endpoint of this study. DSS was calculated from the time of the first PM to the date of death from any cause. Surviving patients were censored on the date of last follow-up. When a two-stage procedure was performed, outcomes were calculated from the date of the first surgery. The time to death was evaluated using Kaplan–Meier curves. The association between variables and survival was analyzed using the log-rank test. The variables that were significant in the univariate analyses were evaluated by multivariate analysis using a Cox proportional hazards regression model. All statistical analyses were performed using SPSS for Windows version 23.0 (Chicago, IL, USA) and *p* < 0.05 was considered statistically significant.

## 3. Results

### 3.1. Patient Characteristics

We identified 95 patients who underwent curative-intent surgery for pulmonary metastases from STSs during the study period. We excluded 26 patients whose primary lesions were in parenchymal organs (*n* = 13), mediastinum (*n* = 1), retroperitoneum (*n* = 9), and head and neck (*n* = 3) and three patients who underwent initial PM at another hospital. Finally, 66 patients were analyzed. The patient demographics and tumor characteristics are listed in Table 1. The median age of the patients was 49 years (range, 17–82 years). The median DFI was 17 months (range, 0–105 months). Histopathological diagnosis was based on the latest classification and synovial sarcoma was the most common histological subtype (24%, *n* = 16), followed by undifferentiated pleomorphic sarcoma (UPS) and liposarcoma (15%, *n* = 10), myxofibrosarcoma (11%, *n* = 7), and leiomyosarcoma (9%, *n* = 6).

### 3.2. Surgical Information and Perioperative Complications

Most patients considered to meet the criteria for PM had a unilateral lesion (88%, *n* = 58) and a solitary pulmonary nodule (59%, *n* = 39). After multidisciplinary discussion, PM was selected for eight patients with bilateral lesions. The median number of lung metastases resected was one (1–12). The median maximum diameter of the lung tumors was 15 (1–60) mm. At the time of pulmonary resection, most patients underwent wedge resection (82%, *n* = 54), followed by segmentectomy (11%, *n* = 7) and lobectomy (7.6%, *n* = 5). Only two patients underwent two-stage PM. Curative resection was obtained in most cases (96%, *n* = 63). The three R2 cases all had intraoperative findings of suspected pleural dissemination, which was proven histologically. Twenty (30%) and four (6%) patients received chemotherapy before and after PM, respectively. Perioperative complications occurred in two patients (3%), one with prolonged air leakage and one with postoperative hemothorax. Both improved with conservative treatment, and no major complications (Clavien–Dindo Classification grade 3/4) occurred. The median postoperative hospital stay was 3 (2–13) days. Perioperative chemotherapy was administered in 20 patients (30%) preoperatively and in four patients (6%) postoperatively. Overall, 68% of patients did not receive perioperative chemotherapy.

### 3.3. Disease-Specific Survival and Prognostic Factors

**Figure 1:**
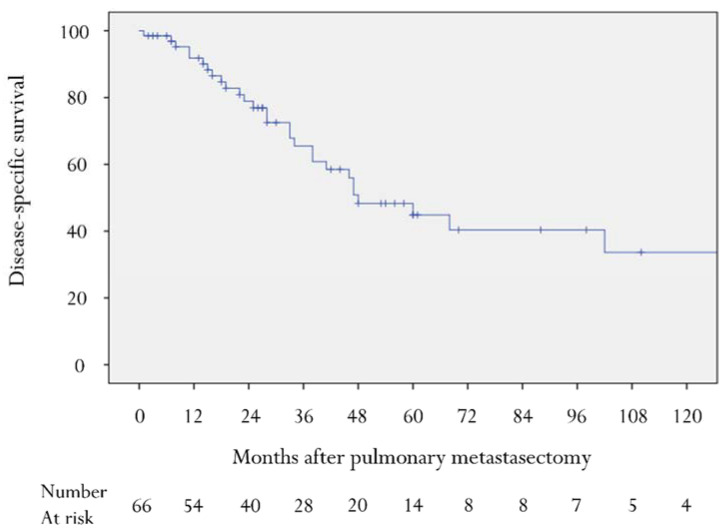
Kaplan–Meier disease-specific survival curve after pulmonary metastasectomy.

In this cohort, the median follow-up was 28 (range, 1–211) months. Median DSS after PM was 48 months (95% confidence interval 29.4–66.6 months) and the 5-year DSS rate was 45% (Figure 1). The relationship between DSS and clinicopathological variables is shown in Table 2. Univariate analysis to determine factors influencing poor DSS identified: DFI (<12 months); primary histology (UPS vs. non-UPS); number of lung metastases (multiple); size of the largest lung lesion (≥20 mm); non-curative resection. Multivariate analysis of prognostic factors is described in Table 3. DFI (<12 months), size of the largest lung lesion (≥20 mm), and non-curative resection were independent prognostic factors, while primary histology and number of lung metastases were not significant.

## 4. Discussion

We reported the results of PM for STSs originating from the trunk wall and extremities. STS is a rare cancer, and its histological types and sites of origin are variable. These issues may make the clinical analysis of STS more difficult. There have been several studies on PM for sarcoma, some of which have analyzed osteosarcoma and uterine sarcoma at the same time, whose prognosis and treatment strategies differ from those of STSs coming from the trunk wall and extremities. For these reasons, we included only STSs of the trunk wall and extremities in this analysis. There are few reports on PM of STSs arising in the trunk and extremities. Kawamoto et al. analyzed 98 cases of pulmonary metastases of STS arising in the trunk and extremities, including those without PM [19]. The median OS for resected and non-resected cases was 38.9 and 10.5 months, respectively. On multivariate analysis, PM was the only prognostic factor. Additionally, another three studies have examined prognostic factors for PM of STS arising in the trunk and extremities. In 1993, Gadd et al. studied 135 pulmonary metastases of STS of extremity origin, including non-operative cases, and found that complete resection was a prognostic factor [3]. In 1995, Choong et al. reported 274 cases of PM of STSs of trunk wall and extremity origin, with a 5-year survival rate of 40% [14]. Multivariate analysis showed that poor prognostic factors were DFI ≤ 18 months, multiple metastases, and metastases > 20 mm in greatest diameter. In 1995, 23 cases of PM of STSs arising in the trunk and extremities experienced at our institution between 1970 and 1992 were reported, with a 5-year survival rate of 32% [15]. The analysis showed the following poor prognostic factors: short DFI, extended chest wall resection, and non-curative resection. However, all these studies are >25 years old, and the prognostic factors for PM need to be confirmed with the current STS classification, which has changed gradually because of the emergence of molecular-based histological subtypes [22].

In this study, short-term DFI, large lung lesions, and non-curative resection were identified as poor prognostic factors for PM of STS. As many other studies have shown [1,8,9,12,14,15,17,21], DFI was one of the important prognostic factors for survival after PM for STSs in this study. Among the literature reviewed on PM for STSs, we could find only one study that found DFI not to be a prognostic factor [4]. It is generally believed that a short DFI indicates that the tumor is highly biologically invasive [2]. Recurrence within 12 months is strongly prognostic and should address the need for strict follow-up procedures [28]. Complete resection has also been reported as an important factor in several studies [8,10,12,15]. In contrast, Chudgar et al., who analyzed the largest number of eligible patients, did not show a benefit of complete resection [1]. The authors mentioned that the small percentage of incomplete resections in eligible patients (R1 = 3%, R2 = 6%) may have resulted in inaccurate results. In the present study, the maximum diameter of pulmonary metastasis of ≥20 mm was a poor prognostic factor. Results from previous studies are still inconclusive as to whether the maximum size was a prognostic factor [8,11,14]. Choong et al. reported that the maximum diameter of pulmonary metastases > 20 mm was a significant prognostic factor in their multivariate analysis, as in the present study [14]. Tumor diameter at the time of diagnosis of pulmonary metastasis may reflect tumor proliferative potential if appropriate surveillance is performed during treatment for STS. In this study, the outcome of multiple pulmonary metastases was significantly worse in univariate analysis, but not in multivariate analysis. The number of metastases as a prognostic factor is still controversial, as is the maximum diameter of the metastases. Several studies have reported the number of lung metastases as a significant prognostic factor [1,10,19], but some have found no significant difference, as in the current study [2,3,4,9]. We have operated mainly on unilateral oligo-metastases. However, based on the results of the present study, if complete resection is deemed possible, aggressive resection may be necessary in the future regardless of the number of metastases.

In the current study, UPS had a poorer prognosis for overall survival in the univariate analysis, but this was not significant in the multivariate analysis. We did not find any studies that identified UPS as a poor prognostic factor after PM of STSs. However, we did find one report that identified MFH as a poor prognostic factor after PM of STSs arising in the trunk wall and extremities in a univariate analysis [14]. In a retrospective study of STS with metastases from initial diagnosis using the Bone and Soft Tissue Tumor Registry in Japan, Zhang et al. reported that the prognosis of patients with UPS was significantly worse than those with other histological subtypes, even after adjusting for age at initial diagnosis, tumor size, and other factors [29]. This finding suggests that the UPS subtype may be more aggressive than others. However, it is difficult to make definitive conclusions because of the limitations of retrospective studies with small numbers of cases. Future multicenter studies of each histological subtype would be desirable.

As in previous studies [1,8,16,21], this study showed no significant difference in survival related to the type of lung resection. Compared with resection of primary lung cancer, anatomic resection may not need to be considered for pulmonary metastases of STSs. Additionally, repeated PM for sarcoma has been shown to be beneficial for survival [4,9,13,21,30,31]. To preserve lung function as much as possible wedge resection is considered suitable for this purpose. However, because complete resection with a negative margin is of utmost importance, anatomic resection is also required depending on the site of pulmonary metastasis. With regard to surgical safety, no serious complications of Clavien–Dindo Classification grade 3/4 were observed in this study. The safety of PM for sarcoma has been reported in several studies [4,32]. Moreover, recent advances in surgical resection have made more minimally invasive and parenchyma-sparing techniques such as ablation therapy and laser resection possible [33,34]. The utility of these techniques versus conventional wide wedge resection has not been established, but they may be useful as an approach for resectable bilateral multiple metastases.

In the present study, there was no evidence for the efficacy of perioperative chemotherapy, nor has it been demonstrated in previous studies [1,8,10,11,14,16,20,21]. Interestingly, our institution reported that the 5-year survival rates with complete resection in a previous study of 23 patients who underwent PM of STSs between 1970 and 1992 was 51.6%. In contrast, in the present study from 2007 to 2021, the 5-year disease-specific survival rates with complete resection were 46%. Furthermore, Schur et al. reported a median survival of 45.3 months in a cohort of 46 patients who underwent PM of STSs between 2003 and 2013, which is comparable to 48 months in the present study [9]. Although all these studies had small sample sizes, were retrospective, and cannot be conclusive, it appears that there has been no significant advance in the prognosis of PM of STS over the past 30 years. One reason for this may be that the efficacy of perioperative chemotherapy for PM of STS has changed little over the same period. However, it is difficult to determine to the efficacy of chemotherapy in this study because 68% of cases did not receive perioperative chemotherapy. The low perioperative chemotherapy rate may be because of the heterogeneity of sarcomas, and local treatment tended to be favored for pulmonary metastases of primary tumors that were less sensitive to chemotherapy. In recent years, newer agents, including molecular targeted drugs, have emerged for the treatment of STS, and we began using them in clinical practice in 2012. It is hoped that further research will clarify their proper use and improve survival. Treatment strategies for tumors that are less sensitive to chemotherapeutic agents are also a future challenge. Ohnstad et al. reported improved progression-free survival in patients with PM of STSs in the preoperative chemotherapy group with a good histological response compared with the group with the poor response [20]. Similarly, ineffective preoperative chemotherapy for pulmonary metastases of STS has been reported to have a worse prognosis [1,10]. These findings suggest that more personalized perioperative chemotherapy may improve outcomes. In an example of personalized chemotherapy, Young et al. showed that doxorubicin–ifosfamide combination therapy is appropriate for patients under 60 years of age with a performance status of 0 or 1 and with poorly differentiated grade III tumors, including UPS [35]. The accumulation of such knowledge may be necessary to improve the outcome of chemotherapy for metastases of heterogeneous STSs. Regarding prognostic factors for PM of STSs, a recent retrospective study utilizing a US multi-institutional database reported that age ≥ 55 years, retroperitoneal primary, R1 resection of primary, and multiple lung metastases (≥2) were associated with decreased OS, but perioperative chemotherapy was not [16]. In this context, prospective multicenter studies of the impact of perioperative chemotherapy on each histologic subtype should be planned.

There were several limitations to this study. First, this was a single-center, retrospective study, and the existence of selection bias cannot be ignored. In particular, PM tended to be performed for unilateral oligo-metastases in the present study, with fewer cases treated with perioperative chemotherapy. Second, this study did not examine patients who did not undergo surgery for pulmonary metastases. Because there was no control group with which to compare outcomes, it was not possible to directly evaluate patients who received PM versus those who did not receive PM. Third, many histological types were analyzed simultaneously in the present study. Fourth, in our analysis, because of the relatively short median follow-up period of 28 months, many cases were censored. Various future multicenter studies on PM of STSs could overcome the rare and heterogeneous nature of STSs.

## 5. Conclusions

We investigated the results of PM of STSs arising in the trunk wall and extremities based on the diagnoses of the latest STS classification. Disease-specific survival was comparable to previous studies, demonstrating the effectiveness of PM of STSs. Short-term DFI, large lung lesion, and non-curative resection were identified as poor prognostic factors, but the number of metastases was not. Unlike many other cancers, aggressive resection may improve the prognosis for pulmonary metastases of STSs that are resectable and have no lesions outside the lungs. Future multicenter trials will be warranted to investigate the significance of PM and appropriate chemotherapy for each histological subtype.

## Figures and Tables

**Table 1:** Clinicopathological Characteristics of Patients.

Variable	Patients (*n* = 66) (%)
**Age (years, median/range)**	49 (17–82)
**Sex**	
Male	38 (58)
Female	28 (42)
**Disease-free interval (months, median/range)**	17 (0–105)
**Primary histology**	
Synovial sarcoma	16 (24)
Undifferentiated pleomorphic sarcoma	10 (15)
Liposarcoma	10 (15)
Dedifferentiated liposarcoma	5 (8)
Pleomorphic liposarcoma	3 (5)
Myxoid liposarcoma	2 (3)
Myxofibrosarcoma	7 (11)
Leiomyosarcoma	6 (9)
Dermatofibrosarcoma protuberans	5 (8)
Ewing sarcoma	3 (5)
CIC-rearranged sarcoma	2 (3)
Malignant peripheral nerve sheath tumor	2 (3)
Sarcoma with BCOR genetic alternations	1 (2)
Epithelioid sarcoma	1 (2)
Extraskeletal osteosarcoma	1 (2)
Alveolar soft part sarcoma	1 (2)
Angiosarcoma	1 (2)
**Primary site**	
Trunk wall	34 (52)
Extremities	32 (48)
**Distribution of lung metastasis**	
Unilateral	58 (88)
Bilateral	8 (12)
**Number of lung metastasis (median/range)**	1 (1–12)
Solitary	39 (59)
Multiple	27 (41)
**Size of largest lung lesion (mm, median/range)**	15 (1–60)
**Type of pulmonary resection**	
Wide wedge resection	54 (82)
Segmentectomy	7 (11)
Lobectomy	5 (8)
**Curative resection**	
R0	63 (96)
R1	0 (0)
R2	3 (5)
**Chemotherapy**	
None	45 (68)
Preoperative	20 (30)
Postoperative	4 (6)
Both	3 (5)

**Table 2:** Univariate Analyses of the Factors Associated with Disease-specific Survival after Pulmonary Metastasectomy.

Variable	*n*	5-Year Survival (%)	*p*
**Age (years)**			
<50	35	50	0.23
≥50	31	37	
**Sex**			
Male	38	53	0.75
Female	28	28	
**Disease-free interval**			
<12 months	22	23	0.001
≥12 months	44	55	
**Primary histology**			
SS	16	36	0.765
Non-SS	50	50	
UPS	10	0	0.001
Non-UPS	56	51	
Liposarcoma	10	57	0.95
Non-liposarcoma	56	44	
MFS	7	50	0.729
Non-MFS	59	44	
Leiomyosarcoma	6	38	0.369
Non-leiomyosarcoma	60	45	
**Primary site**			
Trunk wall	34	47	0.40
Extremities	32	44	
**Size of primary lesion**			
<10 cm	42	47	0.49
≥10 cm	24	48	
**Distribution of lung metastasis**			
Unilateral	58	44	0.536
Bilateral	8	50	
**Number of lung metastases**			
Solitary	39	59	0.047
Multiple	27	27	
**Size of largest lung lesion**			
<20 mm	48	57	<0.001
≥20 mm	18	10	
**Type of pulmonary resection**			
Partial	44	51	0.113
Anatomical	12	21	
**Curative resection**			
Yes	63	46	<0.001
No	3	0	
**Preoperative chemotherapy**			
Yes	20	24	0.051
No	46	54	
**Postoperative chemotherapy**			
Yes	4	25	0.315
No	62	46	

SS: synovial sarcoma; UPS: undifferentiated pleomorphic sarcoma; MFS: myxofibrosarcoma.

**Table 3:** Multivariate Analyses of Factors Associated with Disease-specific Survival after Pulmonary Metastasectomy.

Variable	HR	95% CI	*p*
Disease-free interval (<12 months)	3.544	1.422–8.832	0.007
UPS	2.882	0.858–9.684	0.087
Number of lung metastasis (multiple)	1.195	0.496–2.877	0.691
Size of largest lung lesion (≥20 mm)	3.159	1.117–8.930	0.03
Non-curative resection	12.161	2.011–74.817	0.007

HR: hazard ratio; CI: confidence interval; UPS: undifferentiated pleomorphic sarcoma.

## Data Availability

The data presented in this study are available on request from the corresponding author. The data are not publicly available due to ethical reasons.
